# Digital-Based Interventions for Complex Post-Traumatic Stress Disorder: A Systematic Literature Review

**DOI:** 10.1177/15248380241238760

**Published:** 2024-03-27

**Authors:** Meg Blackie, Kathleen De Boer, Liz Seabrook, Glen Bates, Maja Nedeljkovic

**Affiliations:** 1 Swinburne University of Technology, Hawthorn, VIC, Australia

**Keywords:** PTSD, Hx of child abuse, treatment, intervention/treatment, domestic violence, treatment/intervention, child abuse

## Abstract

Research has shown that complex post-traumatic stress disorder (cPTSD) differs from post-traumatic stress disorder (PTSD) on core symptoms relating to the individual’s sense of self, and this has driven the need for treatment approaches to address these specific features of cPTSD. The COVID-19 pandemic has led to the increased use of digital-based interventions (DBIs) to treat mental illnesses, including trauma-related disorders. However, while evidence for the use of DBIs for PTSD has previously been synthesized, the current review is the first synthesis of research on the use of DBIs for cPTSD. A systematic search of Scopus, PsychINFO, and EBSCOhost was conducted, using search terms targeting “cPTSD” and “DBIs,” to identify research on the use of DBIs to treat cPTSD symptoms. Ten papers were identified, which provided preliminary evidence for the efficacy of DBIs to reduce cPTSD symptoms. Further, DBIs were reported as acceptable by individuals with a history of complex trauma. The paper also provides insight into the therapeutic approaches adopted, digital modalities utilized, safety measures included, and whether/to what degree support was provided. While DBIs show promise for treating cPTSD, there is substantial room for advancement of the empirical evidence base for these approaches. Both clinical and research-based recommendations are provided separately.

Complex post-traumatic stress disorder (cPTSD) is a cluster of symptoms commonly experienced following chronic/prolonged and interpersonal trauma (Ford, 2017). Complex traumas, such as childhood abuse and interpersonal violence, differ from acute traumas that are less interpersonal and more commonly result in post-traumatic stress disorder (PTSD) (e.g., motor vehicle accidents and natural disasters; Ford, 2017). Due to the chronic nature of their trauma, individuals experiencing cPTSD show a disorganization in their sense of self and how they relate to the world, which is not always observed in PTSD (Ford, 2017). Therefore, treatment options are needed that specifically address the unique symptoms of cPTSD. One option for delivering such treatments is digital-based interventions (DBIs). However, although extensive research has been conducted on the use of DBIs for PTSD (e.g., Kuester et al., 2016), there is much less research, and no existing reviews, of the efficacy of these approaches for cPTSD. Therefore, this is the first systematic literature review investigating DBIs for cPTSD.

The COVID-19 global pandemic has made this a critical time to review DBIs for cPTSD for three key reasons. First, mental health clinicians were forced to rapidly transfer their services online during this time, though there was little time to assess the safest and most effective way to do this. Therefore, these approaches must now be reviewed and adjusted where needed. Second, it is predicted that the aftermath of COVID-19 will see a surge in the prevalence of cPTSD, due to increases in the incidence domestic violence and child abuse during this time. Compared to pre-pandemic rates, the rate of domestic violence has increased worldwide (Kourti et al., 2021; Piquero et al., 2021). Additionally, while official rates of child abuse/neglect appeared to decrease, this is likely because children had substantially less contact with those professionals who would usually observe and report the abuse (Campbell, 2020). Third, mental health services were put under increased strain during the pandemic, and DBIs can relieve some of this demand by providing an accessible and effective treatment option (Fairburn & Patel, 2017).

## Complex Post-Traumatic Stress Disorder

cPTSD has only recently been included as a clinical diagnosis, separate to PTSD, within the latest edition of the International Classification of Disease (ICD-11; World Health Organization, 2022). Six core symptoms characterize cPTSD, including the three core symptoms of PTSD: (a) re-experiencing the trauma, as flashbacks or nightmares; (b) avoidance of reminders/triggers of the trauma; and (c) a continuous sense of threat, experienced as hyperarousal or being easily startled. Three additional symptoms then differentiate cPTSD from PTSD: (a) affect dysregulation, presenting as an exaggerated or diminished emotional responses; (b) a negative self-concept, characterized as feelings of failure, worthlessness, guilt, or shame; and (c) interpersonal difficulties with establishing and/or maintaining relationships. These three latter symptoms constitute a disturbance in self-organization (DSO) that is more likely to develop following complex trauma, and they are therefore referred to as the “DSO symptoms” of cPTSD.

Prevalence rates of cPTSD range from 0.5% to 9.3% within community-based samples (Ben-Ezra et al., 2018; de Silva et al., 2021; Maercker et al., 2018; Redican et al., 2022), and from 16% to 56.7% within clinical samples (de Silva et al., 2021; Hyland et al., 2018; Hyland et al., 2017; Karatzias et al., 2016; Murphy et al., 2020). Notably, however, these rates are based on pre-COVID-19 estimates. Further, cPTSD can produce a greater functional impairment compared to PTSD. On average, individuals diagnosed with cPTSD show lower levels of education and self-reported socioeconomic status (SES), have higher rates of unemployment, reduced working capacity, and more comorbid diagnoses, and are more likely to be unmarried and live alone (Brenner et al., 2019; Karatzias et al., 2016). Of note, while being unmarried and living alone is a valid choice for many, in the context of cPTSD, such a lifestyle may not be a choice. Rather, it may reflect a difficulty in building and maintaining close interpersonal relationships, which is a common symptom in people who have experienced chronic interpersonal trauma.

Research on effective treatment approaches for cPTSD was synthesized by Karatzias et al. (2019), who conducted a systematic review and meta-analysis of 51 randomized control trials investigating the effectiveness of in-person interventions for individuals at risk of cPTSD. Their review found that trauma-focused therapies, such as cognitive behavioral therapy (CBT), exposure therapy, and eye movement desensitization and reprocessing therapy (EMDR), were all superior to treatment as usual (TAU) in reducing PTSD symptoms, as well as two DSO symptoms (negative self-concept and interpersonal disturbances). However, there were insufficient data to assess if these therapies effectively improved the third DSO symptom (affect regulation abilities).

### DBIs for Trauma

DBIs can be delivered via a variety of technologies and, as such, the descriptors for DBIs are diverse and use umbrella terms interchangeably (e.g., e-Health, e-Mental Health, e-Therapy), or use technology centric terminology (e.g., internet/online/tele/computer/cyber/ electronic/mobile/web-based/delivered interventions). DBIs also provide consumers with choice in accessing either self-guided or supported interventions. Self-guided interventions can include the support of automated systems (e.g., notifications and prompts), while supported interventions vary in terms of who provides the support, the degree of support provided, and the modality utilized (e.g., video-conferencing, phone calls, text/chat functions, and email) (Andersson, 2016). For this review, DBIs refer to the use of digital technology to remotely deliver interventions based on psychological frameworks; the interventions may be self-guided or supported.

While trauma-focused psychotherapies show promise for treating cPTSD, there can be barriers to accessing these treatments in-person. These include financial limitations, scheduling conflicts, stigma, fear, and limited availability of qualified therapists (due to long treatment waitlists, remote location, or COVID-19 lockdowns) (Lewis et al., 2019; Simon et al., 2019). DBIs provide a means to overcome such barriers; however, a synthesis of research on DBI approaches for this population is currently lacking. In contrast, ten systematic reviews and meta-analyses of DBIs for noncomplex PTSD have been published since 2016 (i.e., Kuester et al., 2016; Lewis et al., 2019; Sander et al., 2020; Sijbrandij et al., 2016; Simblett et al., 2017; Simon et al., 2019; Steubl et al., 2021; Turgoose et al., 2018; Wickersham et al., 2019; Young & Campbell, 2018). Overall, these reviews support the effectiveness of DBIs for PTSD. This suggests that such approaches may also be effective in treating symptoms of cPTSD.

This is the first systematic review of literature, which has investigated DBI approaches for the treatment of cPTSD symptoms specifically. The review aims to improve understanding of the DBIs currently available for cPTSD, and to provide guidance for future researchers and clinicians aiming to improve and implement these approaches. Six research questions (RQs) were addressed: (a) which treatment approaches do DBIs for cPTSD symptoms follow, (b) through which technological modalities are DBIs for cPTSD delivered, (c) do DBIs for cPTSD include human support (d) which symptoms of cPTSD do DBIs target, (e) which processes are employed to ensure the safe, confidential delivery of DBIs for those experiencing symptoms of cPTSD, and (f) what is the efficacy and acceptability of DBIs for cPTSD symptoms?

## Method

This systematic review follows the protocol published through open science (Blackie et al., 2020), which is in-line with the guidelines of the Preferred Reporting Items for Systematic Reviews and Meta-Analysis Protocols (Moher et al., 2015). The review was also registered with PROSPERO, the International Prospective Register of Systematic Reviews (Prospero, 2016) (registration number: CRD42020172921).

### Information Sources and Search Strategy

A systematic search of three databases was conducted to identify literature for this review: Scopus, EBSCOhost, and PsychINFO. Search terms used reflect common synonyms referring to cPTSD and DBIs (see Supplemental Appendix A for the full list of terms entered). The DBI terms were split into “digital” and “intervention” clusters to account for the extensive variability in how these interventions are referred to across the literature. The database searches were limited to the period from 2005 to the present (i.e., at the time of the initial search, February 2021), to ensure that the papers being screened utilized currently available technologies. Within Scopus, the “basic search” function was used, lines 1 and 3 were set to search “All fields,” while line 2 was set to search “Article Title, Abstract and Keywords,” to prevent irrelevant occurrences of these terms being picked up (e.g., “paper published online”). Within EBSCOhost, all databases were selected, the “advanced search” function was used, lines 1 and 3 were set to search “TX All Text,” and line 2 was set to search “AB Abstract.” Within PsychINFO, the “advanced search” function was used, with lines 1 and 3 set to search “Any Field,” and line 2 set to search “Abstract.” The searches were saved in each of the databases, and an alert was set to inform the researchers if any relevant papers were added.

The online screening program Rayyan (Ouzzani et al., 2016) was employed, with two independent reviewers (MB and KDB) screening the compiled literature. The reviewers began with a title and abstract screen, followed by a full text review of all papers flagged as potentially eligible for inclusion. Discrepancies between reviewers were discussed and resolved, with a supervising author (MN) consulted where required. Once screening was complete, additional papers were sourced from within the reference lists of the retained papers, the papers which cited them, and the reference lists of relevant review papers identified in the initial search.

### Eligibility Criteria

#### Participants

As the ICD-11’s diagnosis of cPTSD was only released in 2018, participant eligibility criteria were assessed in two ways. First, if recruited participants met a diagnosis of cPTSD, and second, if recruited participants had a history of exposure to complex trauma (i.e., prolonged and interpersonal). The studies included based on the second method needed to have reported outcome measures for at least one of the DSO symptoms of cPTSD (i.e., the symptoms which differentiate cPTSD from PTSD). Karatzias et al. (2019) followed a similar protocol for assessing the eligibility of studies for inclusion in their systematic review and meta-analysis of in-person treatments for cPTSD. Finally, as research in this specific area was expected to be limited, to maximize the number of papers eligible for review, studies remained eligible for inclusion if their recruited participants were experiencing subclinical symptoms and/or comorbidities.

#### Interventions

Studies were eligible for inclusion if they investigated remotely delivered DBIs, for cPTSD specifically, or for individuals with a history of complex trauma. Interventions could be self-guided or include human guidance/support. Studies were not eligible for inclusion if they were assessing digital media as an organically occurring mental health support (e.g., online support groups), or DBIs not based on a psychological framework (e.g., supportive counseling via videoconference).

#### Outcomes

Eligibility required studies to report the outcome of the DBI on at least one DSO symptom of cPTSD, via either self-report or clinician ratings. Studies were also eligible if they reported acceptability outcomes only (e.g., dropout rates, client satisfaction, and adverse events), as these are directly relevant to part of RQs six.

#### Comparators

Studies were eligible for inclusion if they used any of three types of comparator: (a) waitlist conditions/TAU, (b) alternative active treatment groups, (e.g., in-person interventions or DBIs not based on a psychological framework), or (c) within-group comparisons between pre- and post-intervention on repeated measures.

#### Research Design

Studies utilizing a randomized control trial, controlled trial, or repeated measures research design were eligible for inclusion. Qualitative studies were also eligible, as it was anticipated that they would inform the fifth and sixth RQs s (i.e., risk management strategies and participant feedback). Meta-analyses and literature reviews were excluded from the review. Finally, only peer-reviewed sources published in English were eligible for inclusion.

### Data Extraction

The extracted data fell under four main categories: participant data, intervention related data, outcome data, and study details. The specific points of data extracted under each of these categories were outlined in the review’s protocol (Blackie et al., 2020). All data were extracted independently by one reviewer (MB) into a pre-prepared excel document. A second reviewer (KDB) then chose three of the papers at random and cross checked the extracted data. Any discrepancies were discussed and resolved.

### Data Synthesis

The heterogeneity of the included papers, in both the interventions analyzed and the methods used to do so, was considered too great to allow for the statistical comparisons of a meta-analysis. Therefore, data was synthesized qualitatively in narrative form.

### Risk of Bias Assessment

Risk of bias was assessed using the Quality Assessment Tool for Quantitative Studies (QATQS), developed by the Effective Public Health Practice Project (1998). Two reviewers (MB and KDB) independently used the QATQS to rate each study as being “strong,” “moderate,” or “weak” on six components: (a) selection bias, (b) study design, (c) confounders, (d) blinding, (e) data collection method, and (f) withdrawals and dropouts. Each paper was then assigned a global rating of either “weak” (two or more “weak” ratings), “moderate” (one “weak” rating), or “strong” (no “weak” ratings). The reviewers then cross-checked their ratings, discussed any discrepancies, and agreed on a final rating.

### Amendments to the Protocol

Changes to the protocol written for this systematic review were tracked. Most notably, these changes included an alteration to the extraction method outlined. The protocol stated that all data would be extracted blindly by two reviewers. Instead, however, one reviewer extracted the data (MB) and a second reviewer crosschecked three randomly selected papers (KDB). This decision was made after the first reviewer observed the extensiveness of the extracted data and considered crosschecking a random sample of papers sufficient and more time efficient for the second reviewer. All authors agreed to this change from the protocol. Additionally, two RQs s were added for investigation (questions 2 and 3 outlined above), as the large change in the domain of DBIs during the COVID-19 pandemic (after the protocol was written) highlighted the necessity of addressing these questions relating to modality and human support. Other changes included the addition of one category of data to be extracted (study details), as well as specifications added to the search strategy (i.e., specific functions used in each database).

## Results and Discussion

### Screening Process

The initial database search was conducted on the February 4, 2021 and generated a total of 1,680 results. Following title and abstract screening, 40 papers were selected by either one or both reviewers to undergo full text screening; an overall agreement of 98% was observed at this stage of screening. Of the 40 papers that underwent full text screening, the reviewers agreed on the inclusion of three and the exclusion of 29, leaving eight in conflict: an overall agreement rating of 80%. Disagreement was largely confined to a misunderstanding between the reviewers as to which measures reflected an assessment of the DSO symptoms of cPTSD. Upon discussion, it was agreed that measures of anxiety and depression are not valid measures of the DSO symptoms of cPTSD, because they measure the presence of mood/anxiety disorders, rather than one’s ability to regulate emotional experiences acutely. Therefore, while one of the eight papers in conflict was included, seven were excluded. In summary, the database search identified four papers for inclusion (Brand et al., 2019; Knaevelsrud et al., 2017; Lee et al., 2021; Zehetmair et al., 2020).

**Figure 1.**
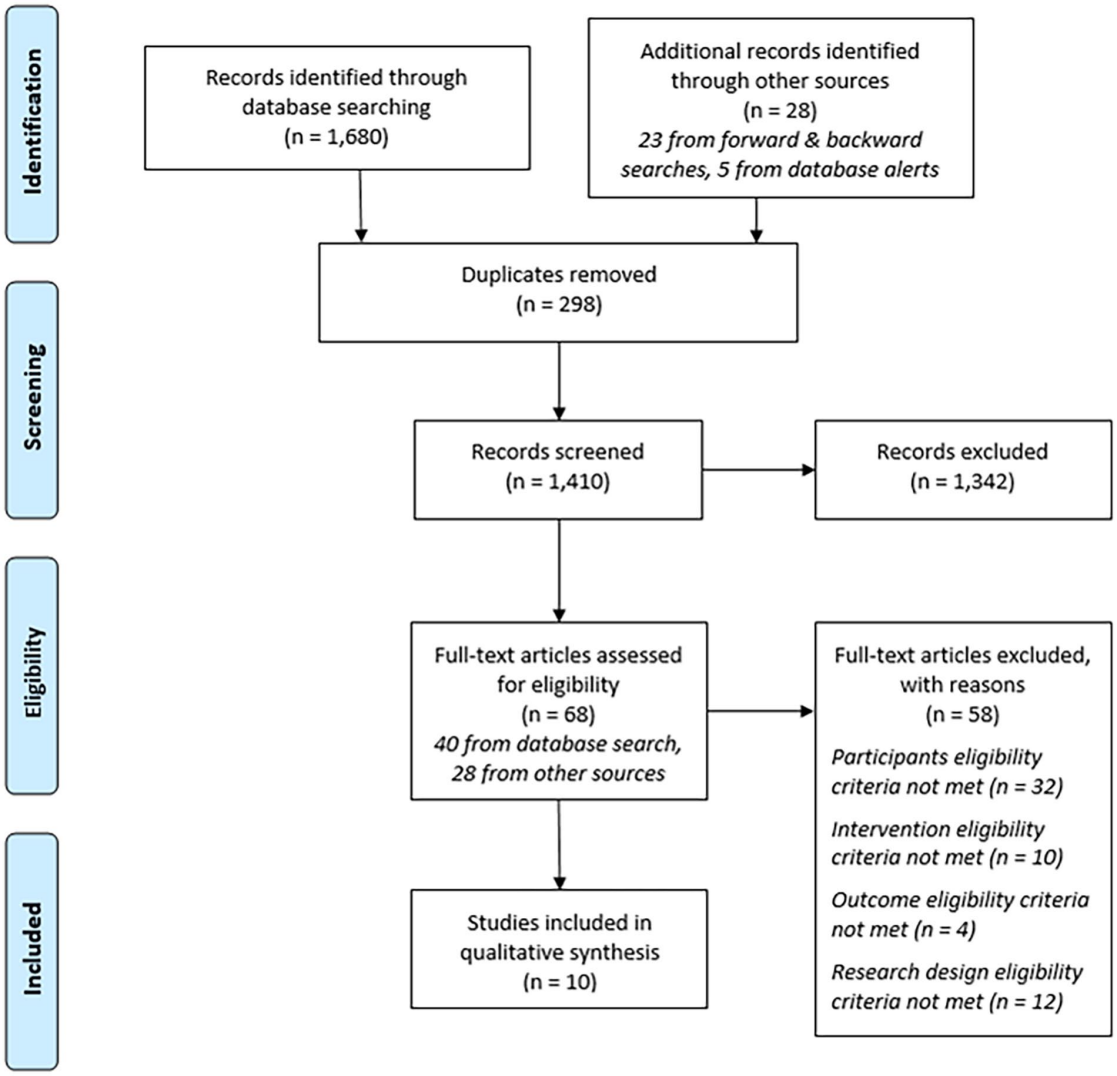
Preferred Reporting Items for Systematic Reviews and Meta-Analysis diagram of the search strategy/screening process; adapted from Moher et al. (2009).

After the initial search, a forward and backward search of the included papers’ citations and reference lists was conducted. This was also done for six relevant review papers identified during the database search (El Morr & Layal, 2020; Kulakli & Shubina, 2020; Lewis et al., 2019; Sijbrandij et al., 2016; Sloan et al., 2011; Stefanopoulou et al., 2019). These searches yielded 23 new papers that were selected for full text screening. Two of these were included in the final review (Fiorillo et al., 2017; Hassija & Gray, 2011). Finally, of the hundreds of papers flagged by the database search alerts, five papers advanced to full text screening, with four selected for inclusion (Bongaerts et al., 2021; Dumarkaite et al., 2021; Robjant et al., 2020; Sabri et al., 2021). This brought the final number of papers included in the review to 10. The screening process is summarized in Figure 1.

### Risk of Bias Assessment

Cross checking the risk of bias ratings between the two reviewers found differences in the overall ratings for all but two papers (Bongaerts et al., 2021; Zehetmair et al., 2020). This was due predominantly to differences in interpretation of the criteria for blinding and was resolved after discussion. The few other differences were oversights and were also resolved after discussion. Thus, the final agreement between the reviewers identified six studies as “strong” (Brand et al., 2019; Dumarkaite et al., 2021; Fiorillo et al., 2017; Knaevelsrud et al., 2017; Lee et al., 2021; Sabri et al., 2021), three as “moderate” (Bongaerts et al., 2021; Hassija & Gray, 2011; Robjant et al., 2020), and one as “weak” (Zehetmair et al., 2020). Although the protocol stipulated that papers receiving a “weak” rating would be excluded from the review, Zehetmair et al.’s paper was retained, as it was a qualitative study and therefore was not fairly assessed by the QATQS. Further, the protocol stipulated that qualitative studies were eligible for inclusion, as the data were anticipated to be rich and relevant to RQs five and six. Thus, all 10 papers were included in the final review.

### Summary of Included Papers

A summary of the included papers is presented in Supplemental Appendix B. Of the 10 papers, half followed a repeated cross-sectional design (*n* = 5), while three presented randomized controlled trials, one a longitudinal follow-up, and one a qualitative study. Date of publication ranged from 2011 to 2021, with most papers being published in the last 3 years (*n* = 7). Four of the studies were conducted in the USA, three in Germany, and one in each of The Netherlands, Lithuania, and South Korea.

Table 1 presents a summary of participant related data across the studies. As anticipated, cPTSD was not measured consistently across the papers (see Table 1, “cPTSD Indication”). Only three papers specifically investigated a DBI for those experiencing cPTSD symptoms (Bongaerts et al., 2021; Dumarkaite et al., 2021; Lee et al., 2021). Six of the remaining papers investigated the use of a DBI for different populations exposed to a form of complex trauma (e.g., refugees, survivors of domestic violence, and childhood abuse). The final paper, by Brand et al. (2019), investigated the use of a DBI for individuals experiencing a dissociative disorder with an implied complex trauma history. This study did not meet the “participant inclusion criteria” for this review, as it neither assessed for cPTSD nor explicitly commented that participants had experienced complex trauma. However, it was included based on the implied complex trauma history (researching a DBI which provides psychoeducation about the impact of trauma to individuals with a dissociative disorder), and because it assessed one of the DSO symptoms as an outcome measure (emotional regulation).

**Table 1.** Summary of Participant Details Across the Included Studies.

	*N*	Age *M*(*SD*)	Gender	Ethnicity	Exclusion	cPTSD Indication
Bongaerts et al. (2021)	6	38.7 (16.07)	Female (4) Male (2)	—	Attempted suicide in the last 3 months	cPTSD measure used: ITQ
Brand et al. (2019)	111 High DES: 71 Low DES: 40	High DES: 43.1 (9.73) Low DES: 41.98 (11.23)	Female (98) Male (12) Trans (1)	96% Caucasian	—	Implied history of complex trauma
Dumarkaite et al. (2021)	70 Ex.: 31 WLC: 39	23.34 (3.11)	Female (61) Male (9)	94% Lithuanian	Current acute psychiatric care Alcohol/drug abuse Ongoing interpersonal violence	cPTSD measure used: ITQ
Fiorillo et al. (2017)	25	39.12 (16)	all female	76% Caucasian	Males Inadequate internet access Emotional abuse only	History of interpersonal trauma
Hassija and Gray (2011)	15	30 (9.25)	all female	86.7% Caucasian	Acutely suicidal or homicidal	Victims of domestic violence and rape
Knaevelsrud et al. (2017)	94 Ex.: 47 WLC: 47	71.4 (4.7)	Female (61) Male (33)	—	Severe depression Suicide risk Alcohol/drug abuse Engaged in other therapy	Experienced a traumatic event/s as a child or adolescent during World War II
Lee et al. (2021)	88 IR: 28 IRO: 31 Control: 29	26.73 (5.58)	Female (59) Male (29)	—	—	Deemed at risk for cPTSD if at least three domains on SIDES-SR were met
Robjant et al. (2020)	14	—	—	Immigrants (Asian, European, or African)	Current suicidality Current severe dissociative symptoms	Migrants or asylum-seekers who identify as BIPOC and/or LGBTQIA+
Sabri et al. (2021)	70	33.8 (7.76)	all female	Black immigrant women (88% African)	—	Immigrant women who are survivors of cumulative trauma
Zehetmair et al. (2020)	42	33.67 (8.3)	Female (17) Male (25)	55% Middle Eastern 29% Sub-Sahara African	Substance use Current psychosis	Refugees seeking asylum

*Note.* BIPOC = Black Indigenous People of Color; DES = Dissociative Experiences Scale (Carlson & Putnam, 1993); Ex. = experimental group; IR = Imagery Rescripting group; IRO = Imagery Rescripting Other group; ITQ = International Trauma Questionnaire (Cloitre et al., 2018); LGBTQIA+ = Lesbian Gay Bisexual Transgender Queer Intersexual Asexual Plus; SIDES-SR = Self-reported Inventory for Disorders of Extreme Stress (Spinazzola, 2019); WLC = waitlist controls.

Reports of severity differed substantially across the papers. Inclusion in this review required that participants in a study had been experiencing either clinical or subclinical cPTSD symptoms. However, only two papers reported this as part of their eligibility criteria (Bongaerts et al., 2021; Dumarkaite et al., 2021), and one required participants to be at risk of cPTSD (Lee et al., 2021). Six of the other seven papers required participants to have a history of complex trauma and to be experiencing either clinically significant PTSD (Knaevelsrud et al., 2017; Robjant et al., 2020; Zehetmair et al., 2020), PTSD or depression (Sabri et al., 2021), a dissociative disorder (Brand et al., 2019), or report a high level of psychological distress (Fiorillo et al., 2017). The final paper recruited survivors of domestic violence or sexual assault, but did not require any level of clinical diagnosis for inclusion (Hassija & Gray, 2011). As reporting of cPTSD severity was not common among the screened papers, having a history of complex trauma and volunteering to participate in a study aiming to improve symptoms resulting from such trauma was deemed to be sufficiently indicative of at least subclinical cPTSD symptoms.

### Analyses of the Six RQs

#### RQ1: Which Treatment Approaches do DBIs for cPTSD Follow?

Table 2 summarizes information relating to the DBIs investigated in each of the papers, including the therapeutic approach utilized and the duration of the intervention. Six studies delivered an intervention following a psychotherapeutic framework, whereas the remaining four studies (40%) focused on psychoeducation and teaching stabilization techniques (including mindfulness training). Notably, in Karatzias et al.’s (2019) review of in-person treatments for cPTSD, only one of their 51 included studies (2%) focused exclusively on mindfulness/stabilization. This suggests that compared to in-person treatments, DBIs for cPTSD are currently more likely to be used solely to provide initial stabilizations techniques. Thus, while trauma-focused DBIs may be beneficial in providing access to treatment for those cPTSD clients impacted by barriers to in-person therapy, DBIs focused on stabilization and safety may be utilized more generally to ease pressure on mental health care systems. Following this, trauma-focused work could then be delivered in the modality of the client’s choosing (i.e., via in-person approaches, remotely via DBIs, or through a blended approach).

**Table 2.** Summary of DBIs Investigated Across the Included Studies.

	Treatment Approach	Modality	Support	Duration	Number of Sessions	Length of Sessions
Bongaerts et al. (2021)	Prolonged Exposure Therapy, EMDR, psychoeducation, and physical exercise	Videoconferencing	Synchronous support from a rotation of psychologists.	4 days	8 (2 per day)	90 min
Brand et al. (2019)	Psychoeducation	Secure online platform	External support from participants’ psychologist.	Ongoing access	45 videos	5–15 min
Dumarkaite et al. (2021)	Trauma focused mindfulness exercises, with a focus on psychoeducation	Secure online platform	Option for asynchronous support, with a reminder at the end of each recording.	8 weeks	8 audio recordings	2–7 min
Fiorillo et al. (2017)	Acceptance and Commitment Therapy	Secure online platform	Phone call or email prompts to complete modules.	6 weeks	6	60 min
Hassija and Gray (2011)	Trauma focused therapy; either Prolonged Exposure or Cognitive Processing Therapy*	Videoconferencing	Synchronous support.	Mean of 13 weeks	Mean of 13	60–90 min
Knaevelsrud et al. (2017)	Integrative Testimonial Therapy**	Secure online platform	Asynchronous support via written feedback.	6 weeks	11 (2 per week)	45 min
Lee et al. (2021)	Imagery Rescripting Therapy***	Secure online platform	n/a	1 session	1	<30 min
Robjant et al. (2020)	Narrative Exposure Therapy	Videoconferencing	Synchronous support.	13 weeks	8	90 min
Sabri et al. (2021)	Mindfulness-based stress reduction and psychoeducation	Phone & online educational platform	Synchronous support during phone session. Option for support during modules.	4 weeks	4 phone sessions; number of modules unclear	25–35-min phone sessions; length of modules unclear
Zehetmair et al. (2020)	Stabilizing and Guided Imagery Techniques	Audio file transferred to participants’ mobile	In person support during initial session & booster session.	Ongoing access	One audio file; three techniques	Unclear

*Note.* EMDR = Eye Movement Desensitization and Reprocessing Therapy.

* Motivational Interviewing also used to assist participants decide whether to leave an abusive relationship.

** Integrative Testimonial Therapy was developed from components of Narrative Therapy and Cognitive Behavioral Therapy.

*** Imagery Rescripting Therapy “other” was also assessed.

#### RQ2: What Technological Modality do DBIs for cPTSD Utilize?

Nine of the 10 studies required participants to have access to the internet. Zehetmair et al. (2020) was the only study that did not require internet access for the DBI, as it involved transferring audio files to the devices of refugees, who could then play the files offline. Of the studies requiring internet, five delivered treatments through an interactive secure online platform, three made use of videoconferencing technologies, and one used a combination of phone calls and an online educational platform (see Table 2, “Modality”).

The finding that most DBIs required an internet connection is in line with the findings from systematic reviews of DBIs for noncomplex PTSD. For example, Simblett et al. (2017) and Steubl et al. (2021) conducted systematic reviews of DBIs for PTSD (both internet- and non-internet-based) and found that the majority of the included studies investigated interventions, which required an internet connection (97% [*n* = 39] and 81.8% [*n* = 33], respectively). Similarly, while there have been only two additional systematic reviews of DBIs for PTSD, which did not require an internet connection (Sander et al., 2020; Wickersham et al., 2019), there have been an additional six which investigated DBIs that did require an internet connection (Kuester et al., 2016; Lewis et al., 2019; Sijbrandij et al., 2016; Simon et al., 2019; Turgoose et al., 2018; Young & Campbell, 2018). While it is common, therefore, for DBIs to require a stable internet connection, non-internet-based DBIs should be considered for population groups for whom it is common to have no or limited internet access, due to factors such as financial restraints and/or unstable housing arrangements (e.g., refugees and those leaving domestic violence). Such DBIs could focus on stabilization and safety until individuals are able to access trauma-focused therapies, through whichever modality is most suitable for them.

#### RQ3: What Degree of Support is Provided During DBIs for cPTSD?

Therapist support during the DBI was included in 80% of the studies (*n* = 8). Support was provided either by fully qualified or postgraduate psychologists and varied in terms of the frequency of support, and the modality through which it was delivered (see Table 2, “Support”). Of the two papers that did not provide therapist support as part of the DBI, Lee et al.’s (2021) intervention was completely self-directed, whereas Brand et al. (2019) recruited therapist–client dyads, suggesting all participants were accessing external psychological support. Further, only 30% of the studies (*n* = 3) had in-person contact during any stage of the research or treatment. Two conducted the initial assessments in-person, but had no in-person contact during treatment (Bongaerts et al., 2021; Fiorillo et al., 2017). The third included an initial in-person session and a booster session 9 days later, providing participants with a demonstration on how to use the audio files (Zehetmair et al., 2020).

These findings appear to differ from those reported in reviews of DBIs for noncomplex PTSD. Kuester et al. (2016) found in their meta-analysis of internet-based interventions for PTSD, that only 55% (*n* = 20) of the interventions reviewed included therapist support. Kuester et al. reported that, overall, interventions that included support had a larger treatment effect than those which did not, though this difference was not significant. The authors suggested further research focus on this comparison, as previous research has highlighted that therapist support is correlated with better treatment outcomes among individuals experiencing depression and/or anxiety (e.g., Cowpertwait & Clarke (2013)). This recommendation is reiterated here, as there have been no comparisons between supported and nonsupported DBIs for cPTSD. Specifically, it would be informative to know which DBIs for cPTSD benefit from support (i.e., do both trauma-focused and stabilization-focused DBIs benefit from support or could stabilization-focused DBIs be self-guided).

#### RQ4: Which Symptoms of cPTSD are Being Targeted by DBIs?

None of the papers specified which symptoms the DBIs were designed to target in terms of the six core symptoms of cPTSD outlined in the ICD-11. However, most studies (*n* = 9) used a measure of PTSD to assess treatment efficacy. This indicates they were interested in whether the DBI reduced the three core PTSD symptoms of cPTSD. Comparatively, only three studies included a measure of cPTSD (or a DSO symptom) to assess treatment efficacy. This indicates that in most of the studies (*n* = 7), the DBI being investigated was not explicitly designed to target the three DSO symptoms of cPTSD. Of the three studies that included a measure of DSO symptom/s, two used a measure of cPTSD (Bongaerts et al., 2021; Dumarkaite et al., 2021), and the third measured participants’ emotion regulation abilities specifically (Brand et al., 2019).

The recency of the cPTSD diagnosis was important to consider when identifying which cPTSD symptoms the DBIs were designed to target. That is, while most studies did not evaluate changes in DSO symptoms to assess treatment efficacy, looking at the treatment approaches employed from a clinical perspective, it can be assumed that at least some of these symptoms were being targeted. For example, aside from Bongaerts et al. (2021), all of the DBIs investigated included elements of stabilization, which is likely to influence the DSO symptom “disturbances in emotional regulation”. Thus, while currently available DBIs may be targeting the DSO symptoms of cPTSD, due to limitations in measuring cPTSD in most papers, whether they do so is yet to be determined empirically. Karatzias et al.’s (2019) review of in-person treatments for cPTSD identified the same methodological limitation. Both reviews, therefore, highlight the need to include established measures of cPTSD in future research. This will allow for an assessment of the treatment effectiveness of DBIs for all relevant symptoms experienced by this population.

#### RQ5: What Safety Measures are Included in DBIs for cPTSD?

Four of the 10 papers described the safety measures incorporated into their DBIs (Dumarkaite et al., 2021; Fiorillo et al., 2017; Robjant et al., 2020; Sabri et al., 2021). As per the review protocol, the corresponding authors of the six other papers were contacted and asked to provide the details of any safety measures that had been employed. Four authors responded. One confirmed that their DBI included specific safety measures (Knaevelsrud et al., 2017), and three gave explanations for not including any safety measures (Bongaerts et al., 2021; Brand et al., 2019; Hassija & Gray, 2011). Bongaerts et al. (2021) explained that their decision not to include any precautionary stabilization or safety measures was based on their vision and findings from associated research for treating cPTSD. Brand et al. (2019) explained that it was unnecessary for them to include safety measures within their DBI, as it was offered internationally, and participants were required to have the support of an external therapist to monitor safety. Finally, Hassija and Gray (2011) explained that their participants were accessing the intervention from a domestic violence/rape crisis center, where in-person support was available if needed. Due to their nonresponses, information about any safety measures employed by Lee et al. (2021) and Zehetmair et al. (2020) could not be included in this review.

In all, five studies described a total of 10 safety measures that were incorporated into the DBIs: (a) encouraging participants to inform the researchers if safety concerns arose (Fiorillo et al., 2017); (b) confirming the participant’s location each session (Robjant et al., 2020); (c) regularly checking-in with participants regarding safety (Robjant et al., 2020; Sabri et al., 2021); (d) providing participants with crisis support contacts (Dumarkaite et al., 2021; Fiorillo et al., 2017; Knaevelsrud et al., 2017; Sabri et al., 2021); (e) instructing participants not to engage with the DBI at night so that support was available if needed (Knaevelsrud et al., 2017); (f) suggesting participants call someone after each session or engage with a pleasant activity to re-ground themselves (Knaevelsrud et al., 2017); (g) preparing a crisis intervention plan prior to beginning therapeutic work (including for those in abusive relationships) (Dumarkaite et al., 2021; Robjant et al., 2020; Sabri et al., 2021); (h) recording the contact details for a support person who agreed to be close by for every session (Robjant et al., 2020); (i) delivering the DBI through a secure online platform to ensure confidentiality (Knaevelsrud et al., 2017); and (j) contacting dropouts to enquire about safety (Fiorillo et al., 2017). Additionally, six of the 10 studies also attempted to facilitate safety by not accepting into treatment; participants were assessed as either being at risk of suicide (Bongaerts et al., 2021; Fiorillo et al., 2017; Hassija & Gray, 2011; Knaevelsrud et al., 2017; Robjant et al., 2020) or those experiencing ongoing interpersonal violence (Dumarkaite et al., 2021).

Developing effective safety protocols for DBIs available for cPTSD is an important consideration. Although many of the studies reduced risk by not accepting into treatment participants who were at risk of suicide or domestic violence, such individuals are common in clinical settings and require support. Additionally, it is timely to reflect on how safety was managed for this population during the COVID-19 pandemic, and to consider how it can best be facilitated in future DBIs. The 10 safety measures identified above provide guidance on how this can be achieved. Additionally, Brand et al. (2019) demonstrate how safety could be monitored in an internationally offered DBI, by requiring clients to be engaged in regular contact with a personal therapist. Hassija and Gray (2011) applied the same approach for local services lacking therapists trained in trauma-focused therapy, who could utilize the DBI to offer their clients more tailored support. Of course, it will be important to consider and adhere to the legal, regulatory, and accreditation rules, which apply in countries in which the DBI is delivered.

#### RQ6: What is the Efficacy and Acceptability of DBIs for cPTSD?

Table 3 summarizes the outcomes of interest across the studies. Notably, all studies included additional outcome measures (e.g., depression, anxiety, general distress, and quality of life). However, these outcomes were not relevant to the focus of this review and so are not reported—a complete list of additional measures utilized across the included studies is presented in Supplemental Appendix D. For ease of analysis in this section, the findings have been separated into PTSD outcomes, cPTSD/DSO symptom outcomes, and client satisfaction/acceptability outcomes. The specific measures used to assess these outcomes across the different studies are presented in Supplemental Appendix C.

**Table 3.** Summary of Efficacy and Acceptability Outcomes Across the Included Studies.

	Timepoints	PTSD Symptoms	cPTSD/DSO Symptoms	Consumer Satisfaction/Acceptability
Bongaerts et al. (2021)	(1) Pre-treatment (2) Post-treatment (3) 1-month follow-up	• * decrease in PTSD on CAPS-5 (T2 & 3) (*d* = 1.04; 0.92) • * decrease in PTSD on PCL-5 (T2 & 3) (*d* = 0.93; 1.24) • 4/6 no longer met diagnosis (T2 & 3) • 1/6 maintained diagnosis (T2 & 3) • 1/6 developed diagnosis (T2 & 3)	• 1/6 did not meet diagnosis at any T • 3/6 no longer met diagnosis (T2 & 3) • 1/6 maintained diagnosis (T2 & 3) • 1/6 developed a diagnosis (T2 & 3)	• 33% of those invited engaged with the DBI • 0% of those who engaged dropped out • No adverse events reported
Brand et al. (2019)	(1) Baseline (2) 1-year follow-up (3) 2-year follow-up	• Small * improvement (1-year) (*d* = 0.41) • Medium * improvement (2-years) (*d* = 0.65)	• Small * improvement in emotion regulation abilities (1-year) (*d* = 0.36) • Large * improvement in emotion regulation abilities (2-years) (*d* = 0.90)	• Some who were already stabilized dropped out • Educational material reported to be relevant, clear, and useful
Dumarkaite et al. (2021)	(1) Pre-treatment (2) 3-month follow-up	• Decrease in overall PTSD b/g, though not * (*d* = 0.82) • Decrease in intrusions b/g, though not * (*d* = 0.36) • Decrease in avoidance b/g, though not * (*d* = 0.73) • * decrease in hyperarousal b/g (*d* = 0.83)	• * decrease b/g in: - overall cPTSD score (PTSD + DSO) (*d* = 0.39) - overall DSO symptoms (*d* = 0.59) - negative self-concept (*d* = 0.74) - disturbances in relationship (*d* = 0.53) • No * decrease in affect dysregulation (*d* = 0.47)	• Times logged in: ≥1 (100%), <5 (36%), 5–10 (13%), 11–20 (19%), >20 (32%) • Program was useful (80.7%), satisfactory (83.9%), easy to use (93.6%) • Improved mental wellbeing (61.3%) and understanding of themselves (64.6%) • Would recommend to trauma survivors (77.5%)
Fiorillo et al. (2017)	(1) Pre-treatment (2) Post-treatment	• * decrease in symptoms (*d* = 1.06)	—	• 16% dropped out • No adverse events reported • High satisfaction with the program • System usability rated as good
Hassija and Gray (2011)	(1) Pre-treatment (2) Post-treatment	• Large * decrease in symptoms (*d* = 1.17)	—	• High level of satisfaction reported overall • Therapist were helpful (*m* = 4.80/5) and sensitive (*m* = 4.93/5).
Knaevelsrud et al. (2017)	(1) Pre-treatment (2) Post-treatment (3) 3-month follow-up (4) 6-month follow-up (5) 12-month follow-up	• * Decrease b/g in overall PTSD, hyperarousal, and avoidance (*d* = 0.42; 0.47; 0.54) (T1-2) • No * decrease b/g in intrusions (*d* = 0.09) (T1-2) • Treatment effects maintained from T2-5, plus a * decrease in intrusions at T3, 4, and 5 (*d* = 0.47; 0.80; 0.64)	—	• 12.8% of the intervention group dropped out • On average, participants reported: - A high level of working alliance with the therapist - Feeling valued, motivated, & understood by the therapist - Finding it easier to disclose due to having visual anonymity
Lee et al. (2021)	(1) Pre-treatment (2) Post-treatment (3) 1-week follow-up	—	—	• 32% of IR group dropped out • 19% of IRO group dropped out • 17% of control group dropped out
Robjant et al. (2020)	(1) Pre-treatment (2) Halfway point (3) Post-treatment	• Increase in symptoms at T2 for 9/13 • Decrease in symptoms at T3 for all	—	• 100% of participants satisfied with e-NET • Preference for online support reported post-treatment, due to: - Reduced cost & stress associated with travel - Beneficial to be in the comfort of their room
Sabri et al. (2021)	(1) Pre-treatment (2) Post-treatment (3) 3-month follow-up	• * decrease in symptoms (*d* = 0.73)	—	• 91.2% rated motivation to engage 7–10/10; 8% rated 5/10 • Dropout rate: 24% overall, 11% from MBSR, 29% from online modules • Positive feedback reported for MBSR overall • >90% understood the online modules and found them helpful • Ease of disclosing over the phone was commented on
Zehetmair et al. (2020)	(1) Pre-treatment (2) 9-day booster (3) 2-month follow-up	• To be reported in an upcoming paper	—	• 51% of those invited engaged with the DBI • 55% did not attend the booster session • Positive effects reported overall

*Note.* see Supplemental Appendix C for a list of the outcome measures used.

b/g = between groups; CAPS-5 = Clinician-Administered PTSD Scale for DSM-5 (Boeschoten et al., 2018); *d* = Cohen’s *d* effect size; IR = Imagery Rescripting group; IRO = Imagery Rescripting Other group; MBSR = mindfulness-based stress reduction; PCL-5 = Posttraumatic Stress Checklist for DSM-5 (Blevins et al., 2015); T = timepoint.

* Statistically significant.

#### Post-Traumatic Stress Disorder

Eight of the 10 papers reported PTSD outcomes (see Table 3, “PTSD”), and all these studies reported a decrease in PTSD symptoms after completion of the DBI. In six of the eight studies, the decrease was significant. However, in one, the decrease did not reach significance (Dumarkaite et al., 2021), and the final study did not report on the significance of the decrease (Robjant et al., 2020). Dumarkaite et al. (2021) suggested that the failure to achieve a significant decrease in their study may relate to the nature of their sample. That is, their sample included participants with either clinical or subclinical symptom severity, and so Dumarkaite et al. suggest that there may not have been room for significant improvement overall.

Two studies also reported the outcome of the DBI on individual PTSD symptoms (hyperarousal, avoidance, and re-experiencing/intrusions). Dumarkaite et al. (2021) found all three symptom types decreased, though only the decrease in hyperarousal was significant. They suggested that this may be because the DBI being investigated focused on mindfulness, which, by its nature, facilitates avoidance of trauma memories and, therefore, may not be effective in addressing avoidance and re-experiencing symptoms. In line with this, following their trauma-focused DBI, Knaevelsrud et al. (2017) reported decreases in all three symptom types. The decreases in hyperarousal and avoidance were significant post-treatment, and the decrease in trauma-related intrusions was significant at follow-up. Knaevelsrud et al. suggested that the decrease in trauma-related intrusions was delayed until follow-up because their DBI required participants to revisit trauma-memories. This meant that such memories were likely to be easily triggered during treatment and then became less intrusive overtime once processed. Taken together, the findings suggest that trauma-focused approaches are required to effectively to treat the PTSD symptoms of cPTSD with DBIs.

#### Complex Post-Traumatic Stress Disorder

Only two of the 10 studies reported cPTSD outcomes (Bongaerts et al., 2021; Dumarkaite et al., 2021), with a third reporting specific outcomes for one of the DSO symptoms (emotion regulation abilities) (Brand et al., 2019). Looking at cPTSD overall, outcomes were favorable (see Table 3, “cPTSD”). Dumarkaite et al. (2021) reported a significant decrease in cPTSD severity post-treatment with those engaged with the DBI showing greater improvement compared to waitlist controls. Of the six participants in Bongaerts et al.’s (2021) study, four met diagnosis for cPTSD pre-treatment. Three of these participants improved and did not meet diagnosis post-treatment, whereas one maintained their diagnosis. For the two participants who did not meet the criteria for diagnosis pre-treatment, while one maintained this status, the other’s symptoms worsened, and they met a diagnosis post-treatment. This worsening in symptom severity was described as “nonreliable,” though not specified why.

Overall, therapeutic outcomes were also favorable for individual cPTSD DSO symptoms (disturbances in affect regulation, self-concept, and interpersonal relationships). Dumarkaite et al. (2021) found that, compared to waitlist controls, the treatment group had significantly greater improvements in their self-concept and in their interpersonal relationships after engaging with the DBI. However, the groups did not differ on improvement of affect regulation abilities. It was suggested by Dumarkaite et al. that this may have been because their participants reported either clinical or subclinical symptoms prior to treatment. This made it possible that several of their participants may have possessed high affect regulation abilities pre-treatment, and therefore, such abilities were not improved, on average, across the sample. Conversely, Brand et al. (2019) assessed changes in emotion-regulation abilities specifically and found a small but significant improvement in emotion regulation abilities 1 year following the DBI, and a large significant improvement 2 years later. However, as all participants were also receiving external psychological support, it is difficult to evaluate the unique impact of the DBI on emotion regulation abilities. Notably, however, in Karatzias et al.’s (2019) review of in-person treatments for cPTSD, they also found a nonsignificant improvement in affect regulation abilities. Taken together, this suggests that further research with clinical samples is needed, to assess how affect-regulation abilities can be improved for individuals with cPTSD, both in-person and via DBIs.

#### Consumer Satisfaction/Acceptability

All 10 studies included a measure of acceptability/satisfaction. This was assessed via: reports of willingness/motivation to engage with the DBI (*n* = 3), dropout rates (*n* = 8), adverse events (*n* = 2), and/or participant feedback (quantitative or qualitative) (*n* = 8) (see Table 3, “Consumer satisfaction/acceptability”). Each of these measures is discussed separately.

Studies reporting on their participants’ willingness/motivation to engage with the DBIs found that between 33% and 51% of those invited were willing to engage (Bongaerts et al., 2021; Zehetmair et al., 2020). Of those willing to engage, Sabri et al. (2021) reported 91.2% rated their pre-intervention motivation as high, being between 7 and 10/10. This suggests that while motivation to engage with a DBI may be low to moderate initially, motivation may be higher once engaged. Therefore, research investigating how to improve cPTSD clients’ initial motivation to engage with DBIs may be beneficial.

Dropout rates were reported to be between 0% and 32% across the eight studies reporting on this statistic. Notably, Brand et al. (2019) commented that some participants decided to drop out because they had learnt stabilization strategies prior to engaging with the DBI, and therefore did not find it beneficial to continue. Further, Lee et al. (2021)—who evaluated a DBI delivering Imagery Rescripting Therapy—found that dropout rates were 13% lower among participants who were imagining someone else in the rescript of their trauma memories, rather than themselves (19% vs. 32% dropout rates, respectively). Lee et al. suggested that this may be because their participants had lower self-concepts, due to their complex trauma, and so felt more comfortable practicing soothing another person rather than themself. This suggests that future research might investigate whether dropout rates can be reduced by offering stabilization techniques as optional and encouraging participants to practice soothing techniques on an imagined other if they feel more comfortable.

Both studies that monitored for adverse events reported that none occurred during treatment or at the follow-ups. Fiorillo et al. (2017) reported no suicidality, psychiatric hospitalizations, distressed contacts from participants, or symptom worsening among those who did not meet a PTSD diagnosis pre-treatment. They noted, however, that one participant had a deterioration on their PCL-5 and DASS-21-Depression scores. Bongaerts et al. (2021) also reported no reliable symptom worsening or any other adverse events from pre-treatment to either of the follow-ups. However, they reported that one participant met a cPTSD diagnosis post-treatment, who did not meet a diagnosis pre-treatment.

Participant feedback across the studies indicated high levels of satisfaction with the DBIs overall. Two studies reported that their participants found it easier to disclose because they had visual anonymity (Knaevelsrud et al., 2017; Sabri et al., 2021). A third reported that, post-treatment, their participants indicated a preference for online support (Robjant et al., 2020). Finally, in two further studies, participants reported a high level of satisfaction with the therapeutic working alliance. They rated their therapists as helpful, sensitive, understanding, and motivating (Hassija & Gray, 2011; Knaevelsrud et al., 2017).

Sabri et al. (2021) and Zehetmair et al. (2020) reported on participant feedback, which identified four factors that facilitated engagement with the DBIs, as well as six factors that created barriers to engagement. The facilitating factors identified included: (a) the service being confidential/anonymous; (b) having an opportunity to learn more about themselves and available resources/support; (c) being able to take a break to re-focus and manage stress throughout the day; and (d) looking forward to talking to the facilitator/therapist. Conversely, the barriers to engaging with the DBIs included: (a) concerns about the time commitment required and conflicts/changes in scheduling; (b) forgetting, feeling unwell, or an emergency occurring; (c) fear of having to relive their trauma and becoming triggered; (d) feeling sad upon returning to reality after a mindfulness session; (e) being unable to think of a safe place during mindfulness exercises; and (f) facing persistent disruptions due to having children at home. The majority of these barriers are also present when accessing interventions in-person (Kantor et al., 2017). The one barrier unique to DBIs is the difficulty engaging while being interrupted by children at home. Therefore, to facilitate engagement, the client’s external environment should be considered and adjusted where necessary to reduce the occurrence of disruptions.

## Summary Discussion and Recommendations

To date, this is the first review to collate research on the use of DBIs for the treatment of cPTSD symptoms. The 10 included papers provide an initial insight into the RQs investigated (see the “Results and Discussion” section above). While some of the findings reiterate what has already been established from research on DBIs for other mental health conditions, it is important to provide an evidence base to confirm that this research also applies to the cPTSD population. In addition, however, this review has highlighted new insights specific to the design of DBIs for individuals with cPTSD. Currently, for example, DBIs are being used more for stabilization than for trauma-focused work. While this is an appropriate use of these interventions, DBIs targeting the full range of symptoms of cPTSD are lacking. This is a clear gap in the field, as this review provides preliminary evidence that DBIs can effectively treat the DSO symptoms of cPTSD. Importantly, it was also found that clients are more likely to drop out if the DBI focuses on stabilization when they are looking for trauma-focused work. This underscores the need for trauma-focused DBIs. Additionally, this review identified a need for more non-internet-based DBIs for cPTSD, so that refugees and those leaving family violence can access interventions more easily. Directions for future research and clinical practice based on the findings of this review are presented in Table 4.

**Table 4.** Implications and Recommendations for Practice, Policy, and Research.

Research recommendations: (1) Consistent use of terminology when referring to individuals experiencing symptoms resulting from complex trauma would be beneficial. The recent diagnostic label of cPTSD from the ICD-11 is recommended. (2) To review the efficacy of DBIs for all cPTSD symptoms, consistent use of cPTSD specific assessment tools to analyze treatment outcomes is needed. The ITQ is currently available for such use (Cloitre et al., 2018). (3) Direct comparisons of the efficacy and acceptability between self-guided and therapist supported DBIs are needed, including whether this differs between stabilization-focused and trauma-focused interventions. (4) Establishing effective strategies to increase clients’ initial motivation to engage with DBIs may be of benefit; though it should first be assessed whether COVID-19 has negated this, now that many individuals are more familiar and potentially more comfortable with engaging through digital means. (5) Research should assess how affect-regulation abilities can be improved for individuals experiencing cPTSD, via both in-person interventions and DBIs, as a nonsignificant change in affect-regulation abilities was found both in this review and Karatzias et al.’s (2019) review of in-person treatments for cPTSD. (6) Investigation is needed, for whether dropout rates can indeed be reduced by making stabilization work optional and offering clients the option to practice strategies on imagined others initially. (7) Research should consider how diversity factors that impact the digital divide are represented in their samples.
Clinical implications and recommendations: (1) DBIs that do not require internet access should be considered for populations with limited/no access to internet. (2) Clinicians utilizing DBIs are encouraged to establish a personalized safety plan at the beginning of the intervention, as well as employ ongoing monitoring strategies (please see suggestions listed within the paper). (3) To reduce clients’ potential fear of monitoring by another party, any online platforms used should offer secure access, and the client should be informed of this. (4) DBIs delivered internationally, or in locations without therapists trained in trauma-focused therapy, could encourage clients to connect with a local mental health worker, to establish and monitor safety measures. (5) Preliminary evidence from this review suggests that trauma-focused work should be included in any DBI aiming to reduce the “avoidance” and “re-experiencing” symptoms of cPTSD, as purely mindfulness-based interventions may facilitate clients’ avoidance tendencies and therefore not address these symptoms. (6) An appropriate environment to engage with the DBI should be established at intervention onset, assisting the client to arrange an alternative space if there is no home-based option (e.g., at a community outreach program or an appropriate and accessible health clinic). (7) This preliminary review suggests that dropout rates could be reduced by making stabilization work optional and by offering clients with a low self-concept the option to practice strategies on imagined others initially.

*Note.* cPTSD = Complex Post-Traumatic Stress Disorder; DSO = disturbance in self-organization; ICD-11 = International Classification of Disease; ITQ = International Trauma Questionnaire.

There are limitations of the current review to be noted. First, the conclusions that can be drawn from this review are limited by the small number of papers identified for inclusion. Papers were assessed against strict eligibility criteria, stipulating that studies must have investigated a DBI specifically for use with a cPTSD sample, or those with a history of complex trauma. The limited number of eligible papers, therefore, indicates that there is either limited research on this topic, or that available studies have not used consistent terminology. The latter theory is supported by the current authors’ observation that papers often referred to the type of trauma being researched (e.g., “childhood traumatization,” “traumatized refugees”), rather than the overarching terms used in the search strategy for this review. Further, the overarching term “cPTSD” has only recently been accepted into a diagnostic manual (ICD-11), explaining why it has not been used consistently in previous research. A second limitation lies in the reviewed papers, which have included participants with either clinical or subclinical symptoms of cPTSD. As Dumarkaite et al. (2021) noted, this makes it difficult to know the true efficacy of these DBIs within clinical populations. Further, a more statistically powerful meta-analysis of the extracted data was not possible due to a lack of homogeneity in the terminology used, as well as the treatment approaches assessed, and the use of different outcome measures across the 10 included papers.

When discussing DBIs for cPTSD, it is also important to consider how diversity factors influence the digital divide. This refers to how variations in certain factors may increase/decrease the likelihood of someone being able/willing to utilize DBIs (see Lythreatis et al. [2022] for a discussion). The included papers adequately represented diversity in terms of participants’ ages, ethnicity, nationality, and gender (see Table 1), all of which can impact the digital divide. Further, while not reported in the results, the possible impact of SES and education levels also need to be considered. Half of the papers indicated their participants’ SES levels in some way, and across these, diversity was evident. However, diversity was not represented in terms of education. Only three papers reported on this variable, and each reported moderate-to-high levels of education within their samples. Zehetmair et al.’s (2020) paper in particular, however, showed how a DBI can be made accessible for those at a digital divide disadvantage (i.e., refuges with limited access to stable internet).

The COVID-19 pandemic has resulted in a surge of DBI usage, as well as an anticipated rise in the prevalence of cPTSD in the coming years due to increases in domestic violence and child abuse/neglect during this time. Therefore, this is a critical time to review current DBI approaches and to explore how we can ensure the most effective and acceptable use of this treatment modality. The provision of DBIs for cPTSD has the potential to provide increased accessibility to effective treatment options for this population by overcoming many commonly faced barriers (e.g., financial limitations, scheduling conflicts, stigma, fear, and limited availability of qualified therapists). The current review provides early evidence that the use of DBIs for this population is acceptable to clients and shows promise as a treatment modality to effectively address the symptoms specific to cPTSD. However, the review has highlighted that there is substantial space for advancements in the evidence base for treating cPTSD through DBIs. To address this, the review has provided some evidence-based guidance to assist future work directed at improving the use of currently available DBIs for this population and to aid the development of efficacious new DBIs for cPTSD.

## Supplemental Material

sj-docx-1-tva-10.1177_15248380241238760 – Supplemental material for Digital-Based Interventions for Complex Post-Traumatic Stress Disorder: A Systematic Literature Review Supplemental material, sj-docx-1-tva-10.1177_15248380241238760 for Digital-Based Interventions for Complex Post-Traumatic Stress Disorder: A Systematic Literature Review by Meg Blackie, Kathleen De Boer, Liz Seabrook, Glen Bates and Maja Nedeljkovic in Trauma, Violence, & Abuse

sj-docx-2-tva-10.1177_15248380241238760 – Supplemental material for Digital-Based Interventions for Complex Post-Traumatic Stress Disorder: A Systematic Literature Review Supplemental material, sj-docx-2-tva-10.1177_15248380241238760 for Digital-Based Interventions for Complex Post-Traumatic Stress Disorder: A Systematic Literature Review by Meg Blackie, Kathleen De Boer, Liz Seabrook, Glen Bates and Maja Nedeljkovic in Trauma, Violence, & Abuse

sj-docx-3-tva-10.1177_15248380241238760 – Supplemental material for Digital-Based Interventions for Complex Post-Traumatic Stress Disorder: A Systematic Literature Review Supplemental material, sj-docx-3-tva-10.1177_15248380241238760 for Digital-Based Interventions for Complex Post-Traumatic Stress Disorder: A Systematic Literature Review by Meg Blackie, Kathleen De Boer, Liz Seabrook, Glen Bates and Maja Nedeljkovic in Trauma, Violence, & Abuse

sj-docx-4-tva-10.1177_15248380241238760 – Supplemental material for Digital-Based Interventions for Complex Post-Traumatic Stress Disorder: A Systematic Literature Review Supplemental material, sj-docx-4-tva-10.1177_15248380241238760 for Digital-Based Interventions for Complex Post-Traumatic Stress Disorder: A Systematic Literature Review by Meg Blackie, Kathleen De Boer, Liz Seabrook, Glen Bates and Maja Nedeljkovic in Trauma, Violence, & Abuse
