# Supplementary material for: Digital-Based Interventions for Complex Post-Traumatic Stress Disorder: A Systematic Literature Review
Source: Trauma Violence Abuse. 2024 Mar 27;25(4):3115–30. doi: 10.1177/15248380241238760 (PMC11370210; doi:10.1177/15248380241238760)
Supplement: sj-docx-1-tva-10.1177_15248380241238760 – Supplemental material for Digital-Based Interventions for Complex Post-Traumatic Stress Disorder: A Systematic Literature Review [file sj-docx-1-tva-10.1177_15248380241238760.docx]

| **Appendix A.**  *Search Terms Entered into the Databases for the Systematic Literature Search* | |
| --- | --- |
| Line 1 | “complex post-traumatic stress disorder” *OR* “complex posttraumatic stress disorder” *OR* CPTSD *OR “*C-PTSD” *OR* “complex ptsd” *OR* “complex trauma” *OR* DESNOS *OR* “disorders of extreme stress not otherwise specified” *OR “*enduring personality change after catastrophic experience” *OR* EPCACE *OR* “developmental trauma disorder” |
|  | AND |
| Line 2 | online *OR* internet *OR* web *OR* digital *OR* virtual *OR* computer* *OR* email *OR* tele* *OR* cyber *OR* electronic *OR* mobile *OR* eTherapy *OR* “e-Therapy” *OR* eHealth *OR “*e-Health” *OR* “e-Mental” *OR* eMental *OR “*self-help” |
|  | AND |
| Line 3 | therap* *OR* intervention *OR* treatment |
