# Supplementary material for: Digital-Based Interventions for Complex Post-Traumatic Stress Disorder: A Systematic Literature Review
Source: Trauma Violence Abuse. 2024 Mar 27;25(4):3115–30. doi: 10.1177/15248380241238760 (PMC11370210; doi:10.1177/15248380241238760)
Supplement: sj-docx-2-tva-10.1177_15248380241238760 – Supplemental material for Digital-Based Interventions for Complex Post-Traumatic Stress Disorder: A Systematic Literature Review [file sj-docx-2-tva-10.1177_15248380241238760.docx]

| **Appendix B**  *Summary of Included Studies* | | | | |
| --- | --- | --- | --- | --- |
|  | **Article Title** | **Country** | **Study Design** | **Ethics** |
| Bongaerts et al. (2021) | Safety and Effectiveness of Intensive Treatment for Complex PTSD Delivered via Home-Based Telehealth | Netherlands | RCS | Medical ethical exemption granted (IRB00002991, FWA00017598). |
| Brand et al. (2019) | An Online Educational Program for Individuals with Dissociative Disorders and Their Clinicians: 1-Year and 2-Year Follow-Up | USA (international sample) | RLF-U | Received Institutional Review Board approval from Towson University. |
| Dumarkaite et al. (2021) | Effects of Mindfulness‑Based Internet Intervention on ICD‑11 Posttraumatic Stress Disorder and Complex Posttraumatic Stress Disorder Symptoms: A Pilot Randomized Controlled Trial | Lithuania | RCT | Approved by the Vilnius University Psychology Research Ethics Committee (Reference No. 27-02- 2020/36). |
| Fiorillo et al. (2017) | Evaluation of A Web-Based Acceptance and Commitment Therapy Program for Women with Trauma-Related Problems: A Pilot Study | USA | RCS | Received Institutional Review Board approval from the University (unclear which university). |
| Hassija & Gray (2011) | The Effectiveness and Feasibility of Videoconferencing Technology to Provide Evidence-Based Treatment to Rural Domestic Violence and Sexual Assault Populations | USA | RCS | Not reported, though published in a peer-reviewed journal requiring ethics clearance. |
| Knaevelsrud et al. (2017) | Efficacy and Feasibility of a Therapist-Guided Internet-Based Intervention for Older Persons with Childhood Traumatization: A Randomized Controlled Trial | Germany | RCT | Approved by University of Greifswald’s (Germany) ethics committee. Registered in the Australian New Zealand Clinical Trials Registry (ACTRN12608000259347). |
| Lee et al. (2021) | Online Guided Imagery in Traumatic Memory Processing for At-Risk Complex PTSD Adults | South Korea | RCT | Not reported, though published in a peer-reviewed journal requiring ethics clearance. |
| Robjant et al. (2020) | E-NET: Narrative Exposure Therapy Online. The Challenges and Opportunities of Delivering Trauma Therapy Remotely | Germany | RCS | Not reported, though published in a peer-reviewed journal requiring ethics clearance. |
| Sabri et al. (2021) | Development, Feasibility, Acceptability and Preliminary Evaluation of The Internet and Mobile Phone-Based BSHAPE Intervention for Immigrant Survivors of Cumulative Trauma | USA | RCS | Received Institutional Review Board approval from Johns Hopkins University School of Nursing |
| Zehetmair et al. (2020) | Self-Practice of Stabilizing and Guided Imagery Techniques for Traumatized Refugees via Digital Audio Files: Qualitative Study | Germany | Qualitative study | Approved by the University of Heidelberg’s ethics committee (S-640/2016). |
| ***Note:*** RCS (repeated cross-sectional design); RLF-U (repeated longitudinal follow-up); RCT (randomised controlled trial) | | | | |
