# Supplementary material for: Digital-Based Interventions for Complex Post-Traumatic Stress Disorder: A Systematic Literature Review
Source: Trauma Violence Abuse. 2024 Mar 27;25(4):3115–30. doi: 10.1177/15248380241238760 (PMC11370210; doi:10.1177/15248380241238760)
Supplement: sj-docx-3-tva-10.1177_15248380241238760 – Supplemental material for Digital-Based Interventions for Complex Post-Traumatic Stress Disorder: A Systematic Literature Review [file sj-docx-3-tva-10.1177_15248380241238760.docx]

| **Appendix C**  *Summary of Measures used to Assess Outcomes of Interest Across Included Papers* | | | | | | |
| --- | --- | --- | --- | --- | --- | --- |
|  | **PTSD** | **cPTSD** | **Emotion Regulation** | **Self-Concept** | **Quality of Relationships** | **Acceptability** |
| Bongaerts et al. (2021) | CAPS-5  PCL-5 | ITQ | - | - | - | Willingness to engage  Dropouts |
| Brand et al. (2019) | PCL-C | - | DERS | - | - | Dropouts  Participant feedback |
| Dumarkaite et al. (2021) | ITQ | ITQ | - | - | - | Dropouts  User satisfaction  Program usability |
| Fiorillo et al. (2017) | PCL-5 | - | - | - | - | Dropouts  Client Satisfaction Questionnaire  System Usability Scale |
| Hassija & Gray (2011) | PCL-C | - | - | - | - | The Wyoming Telehealth Trauma Clinic Client Satisfaction Scale |
| Knaevelsrud et al. (2017) | PDS | - | - | - | - | Dropouts  Treatment satisfaction questions |
| Lee et al. (2021) | - | - | - | - | - | Dropouts |
| Robjant et al. (2020) | PCL-5 | - | - | - | - | Participant feedback |
| Sabri et al. (2021) | HTQ | - | - | - | - | Motivation to participate  Dropouts  Participant perception and feedback |
| Zehetmair et al. (2020) | PC-PTSD-5 | - | - | - | - | Willingness to engage  Dropouts  Participant feedback |
| ***Note:*** CAPS-5 (Clinician-Administered PTSD Scale for DSM-5; Boeschoten et al., 2018); PCL-5 (Posttraumatic Stress Checklist for DSM-V; Blevins et al., 2015); PCL-C (Posttraumatic Stress Checklist – Civilian Form; Weathers et al., 1993); ITQ (International Trauma Questionnaire; Cloitre et al., 2018); PDS (Post-traumatic Stress Diagnostic Scale; Foa, 1995); HTQ (the Harvard Trauma Questionnaire; Mollica et al., 1992); PC-PTSD-5 (Primary Care PTSD Screen for DSM-5; Prins et al., 2016); DERS (The Difficulties in Emotion Regulation Scale; Gratz & Roemer, 2004). | | | | | | |

**References**

Foa, E. B. (1995). *Posttraumatic stress diagnostic scale*. National Computer Systems.

Gratz, K. L., & Roemer, L. (2004). Multidimensional assessment of emotion regulation and dysregulation: Development, factor structure, and initial validation of the difficulties in emotion regulation scale. *Journal of* *Psychopathology and Behavioral Assessment*, *26*(1), 41–54.

Mollica, R. F., Caspi-Yavin, Y., Bollini, P., Truong, T., Tor, S., & Lavelle, J. (1992). The Harvard Trauma Questionnaire: Validating a cross-cultural instrument for measuring torture, trauma, and posttraumatic stress disorder in Indochinese refugees. *Journal of Nervous and Mental Disease*, *180*(2), 111–116.

Prins, A., Bovin, M. J., Smolenski, D. J., Marx, B. P., Kimerling, R., Jenkins-Guarnieri, M. A., Kaloupek, D. G., Schnurr, P. P., Kaiser, A. P., & Leyva, Y. E. (2016). The primary care PTSD screen for DSM-5 (PC-PTSD-5): Development and evaluation within a veteran primary care sample. *Journal of General Internal Medicine*, 31(10), 1206–1211.

Weathers, F. W., Litz, B. T., Herman, D. S., Huska, J. A., & Keane, T. M. (1993). The PTSD Checklist (PCL): Reliability, validity, and diagnostic utility. International Society Traumatic Stress Studies, 2, 90-92.
