# Supplementary material for: Digital-Based Interventions for Complex Post-Traumatic Stress Disorder: A Systematic Literature Review
Source: Trauma Violence Abuse. 2024 Mar 27;25(4):3115–30. doi: 10.1177/15248380241238760 (PMC11370210; doi:10.1177/15248380241238760)
Supplement: sj-docx-4-tva-10.1177_15248380241238760 – Supplemental material for Digital-Based Interventions for Complex Post-Traumatic Stress Disorder: A Systematic Literature Review [file sj-docx-4-tva-10.1177_15248380241238760.docx]

| **Appendix D**  *Additional Outcome Measures Utilised Across the Included Studies* | | |
| --- | --- | --- |
|  | **Timepoints** | **Measures** |
| Bongaerts et al. (2021) | 1. Pre-treatment 2. Post-treatment 3. 1-month follow-up | - M.I.N.I (Mini International Neuropsychiatric Interview) |
| Brand et al. (2019) | 1. Baseline 2. 1-year follow-up 3. 2-year follow-up | - DES (Dissociative Experiences Scale II) - PITQ-p (Progress in Treatment Questionnaire, patient version) - PITQ-t (Progress in Treatment Questionnaire, therapist version) - Unsafe/Unhealthy Behaviours (NSSI [Non suicidal self-injury], suicidality, and risky behaviour |
| Dumarkaite et al. (2021) | 1. Pre-treatment 2. 3-month follow-up | - PHQ-9 (Positive Mental Health Scale) - GAD-7 (Generalized Anxiety Disorder Scale) - PMH (Patient Health Questionnaire) |
| Fiorillo et al. (2017) | 1. Pre-treatment 2. Post-treatment | - DASS-21 (Depression, Anxiety, & Stress Scale) - GHQ-12 (General Health Questionnaire) - AAQ-II (Acceptance and Action Questionnaire [measure of psychological flexibility]) |
| Hassija & Gray (2011) | 1. Pre-treatment 2. Post-treatment | - CES-D (Centre for Epidemiological Studies Depression Scale) |
| Knaevelsrud et al. (2017) | 1. Pre-treatment 2. Post-treatment 3. 3-month follow-up 4. 6-month follow-up 5. 12-month follow-up | - BSI-18 (Brief Symptom Inventory [depression, anxiety, and somatization assessment]) - GSE (General Self-Efficacy Scale) - EUROHIS-QOL (Quality of Life Assessment) |
| Lee et al. (2021) | 1. Pre-treatment 2. Post-treatment 3. 1-week follow-up | - PANAS (Positive Affect and Negative Affect Schedule) - TAQ (Trauma Appraisal Questionnaire) - SAM (Stress Appraisal Measure) |
| Robjant et al. (2020) | 1. Pre-treatment 2. Halfway point 3. Post-treatment | - Shut-D (Shutdown Dissociation Scale) - C-SSRS (Columbia–Suicide Severity Rating Scale) |
| Sabri et al. (2021) | 1. Pre-treatment 2. Post-treatment 3. 3-month follow-up | - PSS (Perceived Stress Scale) - IPPA (Inventory of Positive Psychological Attitudes [stress management assessment]) - TCSES (Trauma Coping Self-Efficacy Scale) - PPS-R (Personal Progress Scale-Revised [overall empowerment assessment]) - MOVERS (Measure of Victim Empowerment Related to Safety) - PHQ-9 (Patient Health Questionnaire) |
| Zehetmair et al. (2020) | 1. Pre-treatment 2. 9-day booster 3. 2-month follow-up | - PHQ-2 (Patient Health Questionnaire) - GAD-2 (Generalized Anxiety Disorder Scale) - SAM (Self-Assessment Manikin Scale [non-verbal emotional response measure]) - RHS-15 (Refugee Health Screener) |
